# Extracting unrecognized gene relationships from the biomedical literature via matrix factorizations

**DOI:** 10.1186/1471-2105-9-211

**Published:** 2008-04-24

**Authors:** Hyunsoo Kim, Haesun Park, Barry L Drake

**Affiliations:** 1 Harvard-Partners Center for Genetics and Genomics, Brigham and Women's Hospital, Boston, MA 02115, USA; 2 College of Computing, Georgia Institute of Technology, Atlanta, GA 30332, USA

The original article we published [1] lacked adequate attribution and credit to a previously published work [2] (which is cited in the text below as reference 3 as it was numbered that in our original article). Thus, we would like to make the following corrections and we would like to apologize to the author group and the readers: (1) Page 2, add the citation to the caption of Table 1: "The genes considered in the data set [3]. The letters 'A', 'C' and 'D' in brackets show the relation with Alzheimer's disease, cancer, and development, respectively." (2) Page 2, amend the first sentence under heading *Results and discussion *to: "For evaluation of our methods, we used 50 genes related with Alzheimer's disease, cancer, and brain development, which were constructed and defined in [3]." (3) Page 2, remove the *Reelin signaling pathway *and *Alzheimer's disease pathway *sections, and insert sentences: "The detailed descriptions of the Reelin signaling pathway and the Alzheimer's disease pathway can be found in [3]. Table 2 and 3 show genes associated with these pathways and the cosine similarities between a core gene for each pathway (RELN for the Reelin signaling pathway, APP for the Alzheimer's disease pathway) and genes in the full space and the reduced dimensional space obtained from NMF."

## References

[B1] Kim H, Park H, Drake BL (2007). Extracting unrecognized gene relationships from the biomedical literature via matrix factorizations. BMC Bioinformatics.

[B2] Homayouri R, Heinrich K, Wei L, Berry MW (2005). Gene clustering by latent semantic indexing of MEDLINE abstracts. Bioinformatics.

